# Coverage of intermittent prevention treatment with sulphadoxine-pyrimethamine among pregnant women and congenital malaria in Côte d'Ivoire

**DOI:** 10.1186/1475-2875-10-105

**Published:** 2011-04-29

**Authors:** Henriette A Vanga-Bosson, Patrick A Coffie, Serge Kanhon, Caroline Sloan, Firmin Kouakou, Serge P Eholie, Moussa Kone, François Dabis, Hervé Menan, Didier K Ekouevi

**Affiliations:** 1 UFR des Sciences Pharmaceutiques et Biologiques, Abidjan, Côte d'Ivoire; 2 Programme PAC-CI, Abidjan, Côte d'Ivoire; 3 Institut de Santé Publique, Epidémiologie et Développement (ISPED), Université Victor Segalen Bordeaux 2, Bordeaux, France; 4 Centre INSERM U897, Université Victor Segalen Bordeaux 2, Bordeaux, France; 5 Service des Maladies Infectieuses et Tropicales, CHU de Treichville; 6 Service de Gynécologie Obstétrique CHU de Cocody, Abidjan, Côte d'Ivoire; 7 CeDReS, Centre Hospitalier Universitaire Treichville, Abidjan, Côte d'Ivoire

## Abstract

**Background:**

The World Health Organization (WHO) recommends using insecticide-treated mosquito nets (ITNs) and intermittent preventive treatment with sulphadoxine-pyrimethamine (IPT-SP) to prevent malaria in sub-Saharan Africa. Data on IPT-SP coverage and factors associated with placental malaria parasitaemia and low birth weight (LBW) are scarce in Côte d'Ivoire.

**Methods:**

A multicentre, cross-sectional survey was conducted in Côte d'Ivoire from March to September 2008 at six urban and semi-urban antenatal clinics. Standardized forms were used to collect the demographic information and medical histories of women and their offspring. IPT-SP coverage (≥2 doses) as well as placental and congenital malaria prevalence parasitaemia were estimated. Regression logistics were used to study factors associated with placental malaria and LBW (birth weight of alive babies < 2,500 grams).

**Results:**

Overall, 2,044 women with a median age of 24 years were included in this study. Among them 1017 (49.8%) received ≥2 doses of IPT-SP and 694 (34.0%) received one dose. A total of 99 mothers (4.8%) had placental malaria, and of them, four cases of congenital malaria were diagnosed. Factors that protected from maternal placental malaria parasitaemia were the use of one dose (adjusted odds ratio (aOR), 0.32; 95%CI: 0.19-0.55) or ≥2 doses IPT-SP (aOR: 0.18; 95%CI: 0.10-0.32); the use of ITNs (aOR: 0.47; 95%CI: 0.27-0.82). LBW was associated with primigravidity and placental malaria parasitaemia.

**Conclusion:**

IPT-SP decreases the rate of placental malaria parasitaemia and has a strong dose effect. Despite relatively successful IPT-SP coverage in Côte d'Ivoire, substantial commitments from national authorities are urgently required for such public health campaigns. Strategies, such as providing IPT-SP free of charge and directly observing treatment, should be implemented to increase the use of IPT-SP as well as other prophylactic methods.

## Background

Malaria is a global public health issue especially important in Africa, home to more than 70% of all infections worldwide and 243 million new infections in 2008 [1]. The World Health Organization (WHO) estimates that one child dies of malaria every 45 seconds in Africa, accounting for 20% of all childhood deaths in the malaria endemic region [1,2]. Approximately fifty million women become pregnant in malaria-endemic areas each year. Half of these pregnancies are in sub-Saharan African regions where rates of infection with *Plasmodium falciparum *are steady throughout the year [1,2]. Recent studies have shown that the rate of placental malaria varied between 6% and 41%, according to laboratory investigations and type of intervention initiated [3-6]. The presence of *P. falciparum *parasites in intervillous spaces (IVS) resulted in the increase of maternal morbidity, low birth weight (LBW), and preterm delivery rates [7-9]. Moreover, malaria often leads to severe anaemia in pregnant African women [10].

Strategies for controlling malaria during pregnancy in sub-Saharan Africa often include treatment of the disease and resulting anaemia as well as chemoprophylaxis [1,11]. Weekly chloroquine was previously used to prevent malaria [1], but it is no longer administered in this region because of the recent emergence of resistance. Since 2004, the WHO recommends a more effective strategy for preventing malaria during pregnancy, which includes insecticide-treated mosquito nets (ITNs) and intermittent preventive treatment (IPT) [2,12]. IPT for pregnant women consists of two doses of sulphadoxine-pyrimethamine (SP) (1500/75 mg), administered at scheduled clinic visits at least one month apart, starting in the second trimester [2]. HIV-infected pregnant women must take three doses of IPT with SP (IPT-SP) if they do not already take cotrimoxazole prophylaxis to prevent opportunistic infections [2]. However, the proportion of pregnant women who receive ≥two doses of IPT-SP in sub-Saharan Africa remains relatively low, at rates varying between 3% and 66% [1].

Côte d'Ivoire has the highest prevalence of HIV in West Africa, at 4.7% [13]. It is also located in a region where malaria is endemic, with steady rates of *P. falciparum *infection throughout the year. Although Côte d'Ivoire has implemented the WHO recommendations for preventing malaria since 2005, this implementation has never been evaluated and information on the operational effectiveness of IPT-SP is limited. The objective of this study was to evaluate the coverage of IPT-SP, the prevalence of congenital malaria parasitaemia, and to determine the factors associated with placental malaria and LBW in six health facilities in Côte d'Ivoire.

## Methods

### Study sites

A multicenter, cross-sectional survey was conducted in Côte d'Ivoire between March and September 2008 at six randomly selected antenatal facilities providing prevention of mother-to-child transmission of HIV (PMTCT) services. Details of the selection of the facilities have been presented elsewhere [14].

Briefly, the Ministry of Health provided the list of health facilities in southern and central Côte d'Ivoire providing PMTCT services. Overall, 104 facilities were eligible in November 2006. These PMTCT sites were located in three types of settings: 41 were in Abidjan, 25 were in other urban areas, and 38 were in semi-urban areas. Two health facilities from each setting were randomly selected. San Pedro and Bouaké and the two sites in Abidjan are located in urban areas and Grand-Lahou and Sassandra are located in semi-urban areas.

Overall, two sites were located in Abidjan (in the districts of Yopougon and Koumassi), three in the southern coastal region (Grand-Lahou, Sassandra, San Pedro), and one in the centre region (Bouaké, the country's second largest city). All of the health facilities included in this study provided PMTCT services.

The climate of Côte d'Ivoire's coastal and central regions is tropical with four seasons: two dry and two rainy seasons. The dry seasons last from December to April and from August to September. The rainy seasons last from May to July and from October to November. Average temperatures in Abidjan range from 21°C to 33°C and malaria infections occur throughout the entire year. Malaria transmission is endemic to the entire region in Côte d'Ivoire.

### Sample size calculations

The sample size calculation was based on the estimate of a proportion of placental malaria. In the absence of data on the prevalence of malaria in Côte d'Ivoire, data from Ghana which borders Côte d'Ivoire were used. The prevalence of placental malaria after IPT-SP implementation was 15% [15]. With a margin of error of ± 2% using an alpha type-1 error of 5%, at least 1224 pregnant women should be included during the study period, with a minimum of 200 women from each of the six selected sites.

### Study population

Pregnant women who (i) gave birth at any of the six study clinics during the study period, (ii) gave their verbal informed consent, and (iii) donated placentas for blood collection were enrolled.

### Data collection

In the delivery ward, standardized forms were used to collect demographic information (age, location, etc.), medical histories (parity, gravidity, number of antenatal clinic visits, use of anti-malaria prophylaxis and treatment), and the use of ITNs. This information was obtained from the patients' medical charts. Data were collected on the mother's pregnancy and the child's anthropometric characteristics at birth from the maternity register.

### Laboratory investigations

#### Training

Six pharmacy students working on their dissertations at the "Unité de Formation et de Recherche des Sciences Pharmaceutiques d'Abidjan" underwent a two-week training course conducted by one senior biologist (HVB) at the delivery ward at the University of Cocody Teaching Hospital. They performed all laboratory tests during this study period.

#### Peripheral blood samples

Blood samples were collected from the umbilical cord immediately after delivery. Specimens from all consenting women who gave birth to live infants during the study period were obtained. Pharmacy students extracted 5 mL of blood from the discarded placenta/umbilical cord after delivery and placed the sample in a serum-separating tube that was assigned a unique identifier. These blood samples were used to detect malaria parasitaemia in peripheral blood and to test for HIV.

#### Placental blood samples

After the umbilical cord blood samples were collected, the placenta was placed with the maternal side facing upward. After cleaning with sterile saline solution, a healthy paracentric area was incised. Pharmacy students collected 2 mL of placental blood in sterile EDTA tubes using this incision method, which involves making a shallow incision in the maternal side with sterile scissors and collecting the blood that pools from the IVS.

#### Neonatal blood samples

Blood samples were collected two hours after birth from neonates of which their mothers' placentas were infected with *P. falciparum*. Two drops of blood by puncturing the neonate's heel with a sterile lancet were collected.

#### Malaria diagnosis

Rapid Diagnostic Test (RDT) (ACON^® ^Malaria *Plasmodium falciparum*, ACON Laboratories, Inc.) was used to test peripheral and placental blood for malaria parasitaemia. ACON^® ^is a rapid and immunodiagnostic test that detects the presence of histidine-rich-protein 2 antigen (HRP-2) of *P. falciparum *in whole blood. Positive results for RDT were confirmed by examining both thick and thin stained blood smears with light microscopy.

For peripheral blood, thick blood smears were stained with 10% Giemsa for ten minutes. For placental blood, 24 hours after collection, pharmacy students prepared thick placental blood smears and filter paper specimens. Thick blood smears were air-dried and then stained with 2% Giemsa for 30 minutes. Experienced microscopists observed the thick blood smears at 100× magnification (using immersion oil). The density of *P. falciparum *was determined in the peripheral blood by counting the number of asexual parasites in 200 white blood cells (WBCs). When estimating parasite density per μl, a standard WBC concentration of 8,000 WBCs/μl was assumed. Samples were considered to be negative when no parasites were found after counting 500 WBC.

Each slide was read by two microscopists blinded to the other's readings. A third microscopist settled discrepancies between readings. Filter paper specimens were collected on Whatman 5M filter paper and stored in individual sterile plastic envelopes containing desiccant. Parasite density for placental smears was expressed as the percentage of parasitized red blood cells (RBC) divided by the total number of RBC, after counting at least 1,000 RBC.

#### HIV diagnosis

Umbilical cord blood samples were tested for HIV antibodies with the Determine HIV-1/2 Rapid Test (the Determine assay; Abbott Laboratories, Abbott Park, Ill.). Sera reactive by the Determine assay were tested with the Genie II HIV-1/HIV-2^® ^test (Bio-Rad, Marnes-La-Coquette, France), which differentiates between HIV-1 and HIV-2 infections. If the two tests are positive, then women are considered as infected by HIV. If the first test is negative, then women are considered HIV-negative.

### Definitions

IPT-SP coverage was defined as the uptake of ≥2 doses. Maternal malaria parasitaemia was defined as a reactive rapid test confirmed by the presence of asexual parasites (*P. falciparum*) in a thick of peripheral cord blood (peripheral malaria) or in a placental smear (placental malaria). Congenital malaria parasitaemia was defined as the presence of asexual parasites in peripheral blood within the first day of life. Low birth weight (LBW) was defined as birth weight among alive neonates < 2,500 grams and very LBW was defined as < 2,000 grams.

### Statistical analysis

Student's t-test was used to compute medians and interquartile ranges (IQRs) for continuous variables, and the chi-square test to derive percentages for categorical variables. Univariable and multivariable logistical regressions were performed to determine the factors associated with placental malaria parasitaemia and LBW. For LBW, only alive singleton infants were included in the analyses. In the multivariable analysis, the factors associated with the dependant variable (LBW or placental malaria parasitaemia) based on univariable analysis and the known factors associated with the dependant variable in the literature were included. Statistical analyses were performed using Stata^® ^version 10.0 (StataCorp. 2007. Stata Statistical Software: Release 10. College Station, TX: StataCorp LP).

### Ethics statement

This study was approved by the National Ethics Committee of Côte d'Ivoire's Ministry of Health.

## Results

### Study population

Overall, 2,044 women who gave birth at the six selected delivery wards were included. Their median age was 24 years (interquartile range [IQR], 20-30), and 50.1% of patients were younger than 25 years. Almost all women (97.8%) attended ≥1 antenatal care visits during their pregnancy, and 5.4% (110/2019) were HIV-infected. The median gravidity was 2 [IQR, 1-4], 533 (26.1%) women were primigravidae, 492 (24.1%) were secundigravidae, and 1,019 (49.8%) were multigravidae. The median birth weight was 3,000 grams [IQR, 2,700-3,300]. Table 1 describes the characteristics of the study population according to the number of the IPT-SP dose.

**Table 1:** Description of socio-demographic characteristics of pregnant women in delivery ward according to the number IPT-SP doses in Côte d'Ivoire (N = 2 044)

		IPT-SP dose				P Value
			
	Total	0	1	2	≥3	
	(N = 2 044)	(n = 333)	(n = 694)	(n = 821)	(n = 196)	
***Age (years)***						
Median (IQR)	**24 (20-30)**	25 (20-29)	24 (20-30)	25 (20-30)	24 (20-30)	0.819
< 20	**427 (20.9)**	69 (20.7)	155 (22.3)	163 (19.8)	40 (20.4)	0.365
20-24	**598 (29.2)**	92 (27.5)	201 (29.0)	245 (29.8)	60 (30.6)	
25-29	**492 (24.1)**	96 (28.8)	146 (21.0)	204 (24.9)	46 (23.5)	
≥30	**527 (25.8)**	76 (22.8)	192 (27.7)	209 (25.5)	50 (25.5)	

***Gravidity***						
Median (IQR)	**2 (1-4)**	3 (2-4)	3 (1-4)	2 (1-4)	2 (1-4)	0.120
1	**533 (26.1)**	79 (23.8)	175 (25.2)	223 (27.2)	56 (28.6)	0.135
2	**492 (24.1)**	78 (23.4)	167 (24.1)	190 (23.1)	57 (29.1)	
≥3	**1 019 (49.8)**	176 (52.8)	352 (50.7)	408 (49.7)	83 (42.3)	

***Number of ANC visit***						< 0.001
Median (IQR)	**3 (2-4)**	2 (1-3)	2 (1-3)	3 (2-4)	4 (3-5)	< 0.001
0	**46 (2.2)**	41 (12.3)	1 (0.1)	4 (0.5)	0 (0.0)	
1-3	**1 332 (65.2)**	236 (70.9)	570 (83.1)	457 (55.7)	69 (35.2)	
≥4	**666 (32.6)**	56 (16.8)	123 (17.7)	360 (43.8)	127 (64.8)	

Birth weight (grams)*						
Median (IQR)	**3000 (2700-3300)**	3000 (2600-3250)	3000 (2650-3250)	3000 (2700-3300)	3000 (2700-3350)	0.942
< 2500	**207 (10.6)**	35 (11.3)	79 (12.1)	80 (10.1)	13 (6.8)	0.190
< 2000	**35 (1.8)**	5 (1.6)	13 (2.0)	15 (1.9)	2 (1.0)	0.840

***Mothers'HIV status***						
HIV positive	**110 (5.4)**	14 (4.2)	42 (6.1)	43 (5.2)	11 (5.6)	0.090
HIV negative	**1 914 (93.6)**	311 (93.4)	647 (93.2)	771 (93.9)	185 (94.4)	
Not collected	**20 (1.0)**	8 (2.4)	5 (0.7)	7 (0.8)	0 (0.0)	

***Malaria prophylaxis***						
Chloroquine	**11 (0.5)**	11 (3.3)	0 (0.0)	0 (0.0)	0 (0.0)	< 0.001
ITNs	**980 (47.9)**	98 (29.4)	301 (43.4)	532 (64.8)	49 (25.0)	< 0.001
Others (insecticide)	**272 (13.3)**	67 (20.1)	93 (13.4)	73 (8.9)	39 (19.9)	< 0.001

***Location***						< 0.001
Koumassi	**285 (13.9)**	111 (33.3)	104 (15.0)	60 (7.3)	10 (5.1)	
Yopougon	**400 (19.6)**	82 (24.6)	189 (27.2)	113 (13.8)	16 (8.1)	
Grand-Lahou	**428 (20.9)**	35 (10.5)	129 (18.6)	138 (26.8)	126 (64.3)	
Sassandra	**207 (10.1)**	27 (8.1)	67 (9.7)	70 (8.5)	43 (21.9)	
San-Pedro	**354 (17.3)**	27 (8.1)	89 (12.8)	237 (28.9)	1 (0.5)	
Bouaké	**370 (18.1)**	51 (15.3)	116 (16.7)	203 (24.7)	0 (0.0)	

IQR: Interquartile range, ANC: Antenatal Care, IPT-SP: Intermittent Preventive Treatment with Sulfadoxine-Pyrimethamine

*Only for neonates alive (n = 1945)

### IPT-SP coverage

Of the 2,044 pregnant women, 1,711 women (83.7%) received ≥1 dose of IPT-SP as prophylaxis against malaria. The IPT-SP coverage (≥2 doses) was 49.8% (1017/2044) and varied according to the location of the study, the number of ANC visits and the other prophylaxis used (Table 1). According to the study location, the ITP-SP ranged between 24.5% and 67.3% and was lower in the two districts of Abidjan (the capital city) compared to the four other sites (29.1% vs. 60.2%; p < 0.0001). ITP-SP coverage varied significantly according to the number of ANC visits (39.5% for 1-3 ANC visits vs. 73.1% for ≥4 ANC visits; p < 0.0001). No differences were found according to age, gravidity and mother's HIV status (Table 1).

### Maternal malaria parasitaemia

*Plasmodium falciparum *was detected in the peripheral cord blood of 19 women (0.9%) with a median parasitaemia of 164/μl [IQR, 112-540] and in the placenta of 99 women (4.8%) with a median parasitaemia of 780/μl [IQR, 176-3800] (Table 2). Among the 19 mothers with peripheral malaria parasitaemia, six (31.6%) did not receive IPT-SP, seven (36.8%) received one dose and six (31.6%) received ≥two doses. Among those with placental malaria parasitaemia (n = 99), 39 (39.4%) did not receive IPT-SP, 31 (31.3%) received one dose of IPT-SP and 29 (29.3%) received ≥ two doses.

**Table 2:** Prevalence of malaria in pregnant women in antenatal care (N = 2 044)

		IPT-SP dose				
			
	Total (N = 2 044)	0 (n = 333)	1 (n = 694)	2 (n = 821)	≥3 (n = 196)	P value
***Mothers***						
***Peripheral cord blood positive***						
Rapid Malaria test (ACON^®^)	**21 (1.0)**	6 (1.8)	8 (1.2)	6 (0.7)	1 (0.5)	0.358
95% Confidence interval	**[0.64-1.57]**	[0.66 - 3.88]	[0.50-2.26]	[0.27-1.58]	[0.00-2.81]	
Blood smears	**19 (0.9)**	6 (1.8)	7 (1.0)	6 (0.7)	0 (0.0)	0.564
95% Confidence interval	**[0.56-1.44]**	[0.66 - 3.88]	[0.41-2.07]	[0.27-1.58]	[0.00-1.86]	
***Placental blood positives***						
Rapid Malaria test (ACON^®^)	**109 (5.3)**	39 (11.7)	34 (4.9)	27 (3.3)	9 (4.6)	< 0.001
95% Confidence interval	**[4.40-6.40]**	[8.46-15.66]	[3.42-6.78]	[2.18-4.75]	[2.12-8.54]	
Blood smears	**99 (4.8)**	39 (11.7)	31 (4.5)	23 (2.8)	6 (3.1)	0.026
95% Confidence interval	**[3.95 - 5.86]**	[8.46-15.66]	[3.05-6.28]	[1.78 - 4.17]	[1.13-6.54]	

***Neonates***						
Number of available specimens*	**85**	35	26	19	5	
Peripheral blood positives	**4 (4.7)**	2 (5.7)	1 (3.8)	1 (5.3)	0 (0.0)	1.00
95 % Confidence interval	**[1.30-11.61]**	[0.70-19.16]	[0.10-19.64]	[0.13-26.03]	[0.00-52.18]	

* From mothers with placenta blood positive

ε One missing observation

IQR: Interquartile range

IPT-SP: Intermittent Preventive Treatment with Sulfadoxine-Pyrimethamine

ITNs: insecticide-treated mosquito nets

The factors associated with placental malaria parasitaemia in multivariable analysis were: the non-use of IPT-SP or ITNs during pregnancy, primigravidity, and antenatal care in Grand-Lahou or Sassandra (Table 3). A dose effect was found for the association between IPT-SP and placental malaria parasitaemia. The adjusted odds ratios (aORs) were 0.18 (95% CI, 0.10-0.32) for women who received ≥2 doses IPT-SP and 0.32 (95% CI, 0.19-0.55) for those who received one dose, as compared to women who did not receive IPT-SP. Maternal HIV infection was not associated with placental malaria parasitaemia (aOR, 0.25; 95%CI, 0.04-1.42).

**Table 3:** Factors associated with placental malaria: logistic regression model (N = 2 044)

	Placental malaria		Univariate analysis			Multivariate analysis (N = 2 044)		
			
	(+) (n = 99)	(-) (n = 1 945)	OR	95% CI	P value	AOR	95% CI	P value
***Age (years)***								
< 20	30 (30.3)	397 (20.4)	1	-	-	1	-	-
20-24	35 (35.3)	563 (29.0)	0.82	[0.50-1.36]	0.448	1.39	[0.78-2.47]	0.261
25-29	17 (17.2)	475 (24.4)	0.47	[0.26-0.87]	0.016	1.10	[0.49-2.48]	0.812
≥30	17 (17.2)	510 (26.2)	0.44	[0.24-0.81]	0.008	1.29	[0.53-3.13]	0.574
***Gravidity***								
1	45 (45.5)	488 (25.1)	2.67	[1.69-4.22]	< 0.001	2.96	[1.45-6.04]	0.003
2	20 (20.2)	472 (24.3)	1.23	[0.70-2.15]	0.475	1.18	[0.59-2.33]	0.637
≥3	34 (34.3)	985 (50.6)	1	-	-	1	-	-
***Location***								
Koumassi	9 (9.1)	276 (14.2)	1	-	-	1	-	-
Yopougon	13 (13.1)	387 (19.9)	1.03	[0.43-2.44]	0.946	1.43	[0.59-3.47]	0.427
Grand Lahou	25 (25.2)	403 (20.7)	1.90	[1.87-4.14]	0.105	3.01	[1.31-6.92]	0.009
Sassandra	28 (28.3)	179 (9.2)	4.80	[2.21-10.40]	< 0.001	8.54	[3.76-19.41]	< 0.001
San Pedro	8 (8.1)	346 (17.8)	0.71	[0.27-1.86]	0.485	1.99	[0.70-5.65]	0.198
Bouaké	16 (16.2)	354 (18.2)	1.39	[0.60-3.18]	0.442	2.31	[0.98-5.47]	0.056
***Mothers' HIV status***								
HIV positive	3 (3.0)	107 (5.5)	0.11	[0.02-0.55]	0.01	0.25	[0.04-1.42]	0.119
HIV negative	92 (92.9)	1 822 (93.7)	0.20	[0.07-0.62]	0.01	0.39	[0.11-1.38]	0.144
Not collected	4 (4.1)	16 (0.8)	1	-	-	1	-	-
***Use of IPT-SP***								
Yes (≥2 doses)	29 (29.3)	988 (50.8)	0.22	[0.13-0.36]	< 0.001	0.18	[0.10-0.32]	< 0.001
Yes (1 dose)	31 (31.3)	663 (34.1)	0.35	[0.21-0.58]	< 0.001	0.32	[0.19-0.55]	< 0.001
No	39 (39.4)	294 (15.1)	1	-	-	1	-	-
***ITNs***								
Yes	23 (23.2)	957 (49.2)	0.31	[0.19-0.50]	< 0.001	0.47	[0.27-0.82]	0.008
No	76 (76.8)	988 (50.8)	1	-	-	1	-	-

When performing this analysis by dividing the variable of "IPT-SP" into four categories (none, one, two or ≥3 doses), there was a beneficial effect of a third dose: one dose (aOR = 0.32; 95% CI: 0.19-0.56), two doses (aOR = 0.21 95% CI: 0.12-0.39), and three doses (aOR = 0.12 95% CI: 0.05-0.31), as compared to women who never received IPT-SP.

### Congenital malaria parasitaemia

Blood samples were collected two hours after birth from 85 live neonates born to mothers with placental malaria parasitaemia. The presence of *P. falciparum *was detected among four neonates (4.7%; 95% CI, 1.3-11.6%): two mothers did not receive IPT-SP, one received one dose and one mother (25.0%) received ≥ 2 doses. The median parasitaemia value was 186/μl [IQR, 120-514]. All the mothers of infected babies were primigravidae, and none was infected with HIV. The median age of mothers whose infants had congenital malaria was lower than that of mothers whose children did not have congenital malaria (17 years vs. 22 years, p = 0.048).

### Low birth weight

**Figure 1:**
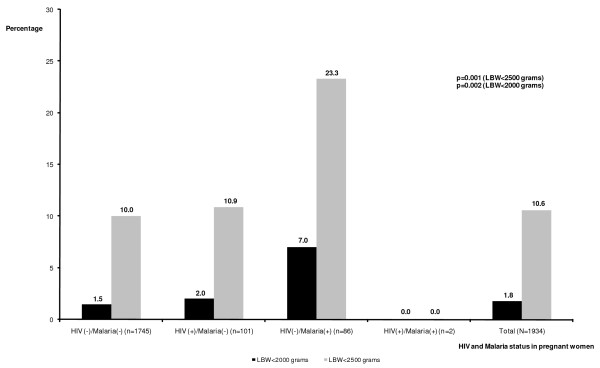
**Low birth weight (LBW) according to HIV and malaria status in pregnant women in delivery wards in Côte d'Ivoire, 2008-2009**.

Overall, 207 neonates (10.6%) out of 1945 had LBW (< 2,500 grams) and 35 (1.8%) had very low birth weight (< 2,000 grams) (Figure 1). The prevalence of LBW was significantly higher among primigravidae (17.5%) than among secundigravidae (9.3%) and multigravidae (7.7%) mothers (p = 0.001). The prevalence of LBW was also significantly higher among babies born to women with placental malaria parasitaemia (22.2%) compared to those born to women without placental malaria parasitaemia (10.1%) (p < 0.001).

Figure 1 presents the proportion of neonates with birth weights < 2,500 grams and < 2,000 grams by maternal HIV and placental malaria status. The prevalence of LBW among neonates born to HIV-uninfected and malaria-infected women (23.3%) was higher than among those born to HIV- and malaria-uninfected women (10.0%) and those born to HIV-infected and malaria-uninfected women (10.9%) (p = 0.001).

The factors associated with LBW in multivariable analysis were mother's age of 20-24 years (aOR = 0.55; 95% CI, 0.36-0.85), primigravidity (aOR = 2.09; 95% CI, 1.27-3.43), and placental malaria parasitaemia (aOR = 2.28; 95%CI, 1.32-3.93) (Table 4). Factors not associated with LBW included maternal HIV infection (aOR = 0.95; 95% CI, 0.18-5.06).

**Table 4:** Factors associated with low birth weight: logistic regression model (N = 1 945)

	Low birth weight (grams)	Univariate analysis			Multivariate analysis (N = 1938)			Multivariate analysis (N = 1938)		
				
	< 2500 (n = 207)	OR	95% CI	P value	AOR	95% CI	P value	AOR	95% CI	P value
***Age (years)***										
< 20 (n = 407)	76 (18.7)	1	-	-	1	-	-	1	-	-
20-24 (n = 575)	49 (8.5)	0.40	[0.28-0.60]	< 0.001	0.55	[0.36-0.85]	0.006	0.58	[0.38-0.89]	0.012
25-29 (n = 469)	44 (9.4)	0.45	[0.30-0.67]	< 0.001	0.79	[0.47-1.34]	0.392	0.83	[0.49-1.40]	0.486
> = 30 (n = 494)	38 (7.7)	0.36	[0.24-0.55]	< 0.001	0.67	[0.37-1.22]	0.192	0.71	[0.39-1.29]	0.265
***Gravidity***										
1 (n = 509)	89 (17.5)	2.55	[1.83-3.55]	< 0.001	2.09	[1.27-3.43]	0.004	2.30	[1.40-3.79]	0.001
2 (n = 471)	44 (9.3)	1.24	[0.84-1.83]	0.279	1.21	[0.77-1.92]	0.407	1.25	[0.79-1.98]	0.332
> = 3 (n = 965)	74 (7.7)	1	-	-	1	-	-	1	-	-
***Location***										
Koumassi (n = 268)	21 (7.8)	1	-	-	1	-	-	1	-	-
Yopougon (n = 388)	50 (12.9)	1.74	[1.02-2.97]	0.043	1.80	[1.03-3.06]	0.037	1.81	[1.05-3.14]	0.033
Grand Lahou (n = 394)	38 (9.6)	1.25	[0.72-2.19]	0.423	1.14	[0.64-2.02]	0.649	1.32	[0.73-2.39]	0.348
Sassandra (n = 187)	23 (12.3)	1.65	[0.88-3.08]	0.116	1.46	[0.77-2.77]	0.244	1.79	[0.94-3.41]	0.077
San Pedro (n = 347)	42 (12.1)	1.62	[0.93-2.81]	0.086	1.58	[0.90-2.77]	0.109	1.79	[1.00-3.22]	0.049
Bouaké (n = 361)	33 (9.1)	1.18	[0.67-2.10]	0.564	1.10	[0.61-1.97]	0.754	1.21	[0.66-2.19]	0.534
***Mothers' HIV status***										
HIV positive (n = 103)	11 (10.7)	0.66	[0.13-3.36]	0.615	0.95	[0.18-5.06]	0.949	0.86	[0.16-4.64]	0.858
HIV negative (n = 1829)	194 (10.6)	0.65	[0.14-2.10]	0.581	0.80	[0.17-3.81]	0.781	0.74	[0.15-3.57]	0.709
Not collected (n = 13)	2 (15.4)	1	-	-	1	-	-	1	-	-
***Malaria status***										
Positive (n = 90)	20 (22.2)	2.55	[1.52-4.28]	< 0.001	2.28	[1.32-3.93]	0.003	-	-	-
Negative (n = 1855)	187 (10.1)	1	-	-	1	-	-	-	-	-
***Sexe***										
Male (n = 986)	102 (10.3)	1	-	-	1	-	-	1	-	-
Female (n = 952)	103 (10.8)	1.05	[0.79-1.40]	0.734	1.02	[0.76-1.38]	0.872	1.0	[0.75-1.35]	0.985
Not mentionned (n = 7)	2 (28.6)	-	-	-	-	-	-	-	-	-
***Use of IPT-SP***										
No (n = 310)	35 (11.3)	1	-	-	-	-	-	1	-	-
1 dose (n = 652)	79 (12.1)	1.08	[0.71-1.65]	0.711	-	-	-	0.98	[0.63-1.53]	0.935
> = 2 doses (n = 983)	93 (9.5)	0.82	[0.54-1.24]	0.348	-	-	-	0.71	[0.46-1.12]	0.146

AOR: Adjusted odds ratio; OR: Odds ratio, 95% CI: 95% Confidence interval

IPT-SP: Intermittent Preventive Treatment with Sulfadoxine-Pyrimethamine

## Discussion

In this study, which was conducted in six antenatal care facilities in Côte d'Ivoire, the coverage of IPT-SP was evaluated among 2,044 women giving birth and their newborns. Overall, only half (49.8%) of the pregnant women received a complete dose of IPT-SP (≥2 doses). The prevalence of placental malaria in mothers was estimated at 4.8% and the prevalence of congenital parasitaemia was 4.7% among infants born to mothers with placental malaria parasitaemia. Factors that protected the mothers from placental malaria parasitaemia were the use of IPT-SP or ITNs during pregnancy and multigravidity. The proportion of babies with LBW was 10.6%, with a larger proportion among babies born to women with placental malaria parasitaemia (22.2%) than among those born to women without placental malaria parasitaemia (10.1%).

Five years after Côte d'Ivoire adopted and implemented the 2004 WHO recommendations for the control of malaria in pregnancies throughout sub-Saharan Africa, the prevalence of placental malaria parasitaemia was lower in this study (4.8%) than those reported in recent African studies in which this prevalence ranged from 10.6% to 20.5% [3,6,15]. Comparison between these studies was difficult. However, several explanations may explain this difference. First, the study site locations were different in the two studies. The previous studies were generally conducted in rural areas [3,6,15] while this study was conducted in six urban and semi-urban areas. Second, the characteristics of study population were different. For example, the proportion of primigravidae was higher in the African studies (ranging from 32.7% to 56.2%) [3,6,15] while it was 26.1% in this study. Third, the proportion of ITNs use was different; it ranged between 8% and 30% in the previous study, but it was 48% in this study.

This study clearly showed a dose-effect relation between IPT-SP use and placental malaria, although the possible influence of selection biases cannot be excluded because this study is an observational study. Indeed, a 82% reduction in placental malaria was found among women who took ≧2 doses of IPT-SP, and a 68% reduction were found among women who took one dose, compared to women who did not receive any IPT-SP. Only a few studies have also demonstrated this dose-effect relation [16-18]; most of them having focused on the effectiveness of IPT-SP in preventing placental malaria parasitaemia in comparison with placebo [19] or weekly chloroquine chemoprophylaxis [20,21]. However, IPT-SP coverage in Africa is generally much lower than the 80% target coverage by 2010, as proposed in 2000 in Abuja [1,22]. In 2007-2008, the percentage of women who received at least two doses of IPT-SP during pregnancy ranged from 3% to 66% [1]. In this study, only 49.8% of pregnant women took complete dose (≥two doses). In recent African studies, the coverage of IPT-SP ranged from 12.5% to 58.8% [15,17,18]. The operational challenges to delivering ≥2 doses of IPT-SP during pregnancy include staff shortages, poor drug supplies, poor access to antenatal care, and improper health worker practices [23-25]. In this study, only half of the women (53.0%) attended antenatal care visits at least three times. A similar observation was found in other African studies in which this proportion ranged from 45.8% to 58.7% [3,4,15]. Therefore, strategies, such as free antenatal care, training health care workers, or providing IPT-SP free of charge and directly observed treatment (DOT), must be implemented or must be encouraged in most African countries to increase the access to antenatal care and IPT-SP use as well as other prophylactic methods such as ITNs. Additional qualitative studies consisting of in-depth interviews of women as well as health care workers should be conducted to determine the existing obstacles to complete IPT-SP coverage.

In this study, the prevalence of congenital malaria was 4.7% among children born to mothers with placental malaria parasitaemia, and all the cases of congenital malaria occurred among adolescent and primigravidae women. A possible explanation for these results is that the immunity women acquire from a first pregnancy affected with malaria helps control the subsequent parasitization of the placenta [26,27]. Generally, the prevalence of congenital malaria in a holoendemic area is globally below 2% and is estimated among all children without regard to their mother's status [28-30]. In this study, infants with congenital malaria were not further followed to observe if they developed symptoms of malaria. In one study conducted in Nigeria, among the 95 neonates with congenital malaria, spontaneous clearance of parasitaemia occurred in 62.1% of neonates before the second day of life and 33.7% were symptomatic within three days of birth [29].

The overall prevalence of LBW was 10.6%, with almost twice as many low-weight babies born to women with placental malaria parasitaemia (22.2%) compared to malaria-uninfected women (10.1%). These findings are consistent with previous studies that report LBW prevalence rates range from 12.4% to 17.3% [6,15,20] and define placental malaria parasitaemia as a predictor of LBW [5,7,31]. In this study, IPT-SP was not associated with a reduction in LBW; however, some studies had highlighted an association [17,18,32]. A comparison between this study and others is difficult to make because the gestational age of women when first dosed of IPT-SP was given was not collected in this study as well as other factors that could explain the occurrence of LBW.

The main strengths of this study were the enrollment of women in six different districts of Côte d'Ivoire, the large sample size, and the assessment of malaria parasitaemia in both mothers and neonates. This study also had several limitations. First, the selected health facilities are only representative of all PMTCT facilities, but may not be representative of all delivery facilities across Côte d'Ivoire. The impact of this limitation was reduced by selecting centers in different regions in Côte d'Ivoire. In addition, the coverage estimates must be interpreted as the best-case scenario because only urban and semi-urban centers were included. Second, because malaria transmission is endemic throughout the entire region in Côte d'Ivoire and because the transmission season lasts 7 to 12 months in all of the regions, based on this study, there is no clear evidence that explains the high prevalence of placental malaria in the coastal sites in comparison to other sites (except for their geographical location). Finally, measurements of the CD4 counts were not performed for the HIV-infected women. The impact of the immune status on the LBW was, therefore, not evaluated.

Additional studies are needed to evaluate the impact of increasing resistance to SP [1] and should also focus on the pharmacokinetics of IPT-SP to determine the optimal dosing interval for pregnant women [33].

In conclusion, international and national advocacy and investments in malaria control have increased substantially in recent years, and there is convincing evidence that, with currently available methods, malaria could shift from a major public health priority to a fairly minor burden for already over-stretched health systems. National health programmes should continue to educate women on the benefits of receiving antenatal care early in their pregnancies and of taking a complete course of IPT-SP. Despite relatively successful IPT-SP coverage in Côte d'Ivoire, substantial commitments on the part of national authorities are urgently required for such public health campaigns. Strategies such as providing IPT-SP free of charge and DOT should be immediately established.

## Competing interests

The authors declare that they have no competing interests.

## Authors' contributions

HAV, PC, HM and DKE conceived of the study, and participated in its design and coordination. PC and DKE performed the statistical analysis. SK participated at the design of the study and its coordination.

CS, PC, HAV, HM, SPE and DKE drafted the manuscript. All authors read and approved the final manuscript.
